# The effect of pre-analytical variables on downstream application and data analysis of human endometrial biopsies

**DOI:** 10.1093/hropen/hoac026

**Published:** 2022-06-13

**Authors:** A Maclean, M Adishesh, L Button, L Richards, R Alnafakh, E Newton, J Drury, D K Hapangama

**Affiliations:** Department of Women's and Children's Health, Institute of Life Course and Medical Sciences, University of Liverpool, Liverpool, UK; Liverpool Women's Hospital NHS Foundation Trust, member of Liverpool Health Partners, Liverpool, UK; Department of Women's and Children's Health, Institute of Life Course and Medical Sciences, University of Liverpool, Liverpool, UK; Liverpool Women's Hospital NHS Foundation Trust, member of Liverpool Health Partners, Liverpool, UK; Department of Women's and Children's Health, Institute of Life Course and Medical Sciences, University of Liverpool, Liverpool, UK; Department of Women's and Children's Health, Institute of Life Course and Medical Sciences, University of Liverpool, Liverpool, UK; Department of Women's and Children's Health, Institute of Life Course and Medical Sciences, University of Liverpool, Liverpool, UK; Department of Women's and Children's Health, Institute of Life Course and Medical Sciences, University of Liverpool, Liverpool, UK; Department of Women's and Children's Health, Institute of Life Course and Medical Sciences, University of Liverpool, Liverpool, UK; Department of Women's and Children's Health, Institute of Life Course and Medical Sciences, University of Liverpool, Liverpool, UK; Liverpool Women's Hospital NHS Foundation Trust, member of Liverpool Health Partners, Liverpool, UK

**Keywords:** pre-analytical variables, endometrial biopsy, endometrial cancer, HIF1α, hypoxia

## Abstract

**STUDY QUESTION:**

What are the effects of pre-analytical variables on the downstream analysis of patient-derived endometrial biopsies?

**SUMMARY ANSWER:**

There are distinct differences in the protein levels of the master regulator of oxygen homeostasis, hypoxia-inducible factor-1-alpha (HIF1α), and the protein and mRNA levels of three related genes, carbonic anhydrase 9 (*CA9*), vascular endothelial growth factor A (*VEGFA*) and progesterone receptor (*PR*) in human endometrial biopsies, depending on the pre-analytical variables: disease status (cancer vs benign), timing of biopsy (pre- vs post-hysterectomy) and type of biopsy (pipelle vs full-thickness).

**WHAT IS KNOWN ALREADY:**

Patient-derived biopsies are vital to endometrial research, but pre-analytical variables relating to their collection may affect downstream analysis, as is evident in other tissues.

**STUDY DESIGN, SIZE, DURATION:**

A prospective observational study including patients undergoing hysterectomy for endometrial cancer (EC) or benign indications was conducted at a large tertiary gynaecological unit in the UK. Endometrial biopsies were obtained at different time points (pre- or post-hysterectomy) using either a pipelle endometrial sampler or as a full-thickness wedge biopsy.

**PARTICIPANTS/MATERIALS, SETTING, METHODS:**

The changes in HIF1α, CA9, VEGFA and PR protein levels were measured by semi-quantitative analysis of immunostaining, and the expression levels of three genes (*CA9*, *VEGFA* and *PR*) were investigated by quantitative real-time PCR, in endometrial biopsies from 43 patients undergoing hysterectomy for EC (n = 22) or benign gynaecological indications (n = 21).

**MAIN RESULTS AND THE ROLE OF CHANCE:**

An increase in HIF1α immunostaining was observed in EC versus benign endometrium (functionalis glands) obtained pre-hysterectomy (*P* < 0.001). An increase in CA9 immunostaining was observed in EC versus benign endometrial functionalis glands at both pre- and post-hysterectomy time points (*P* = 0.03 and *P* = 0.003, respectively). Compared with benign endometrial pipelle samples, EC samples demonstrated increased mRNA expression of *CA9* (pre-hysterectomy *P* < 0.001, post-hysterectomy *P* = 0.008) and *VEGFA* (pre-hysterectomy *P* = 0.004, post-hysterectomy *P* = 0.002). In benign uteri, HIF1α immunoscores (functionalis glands, *P* = 0.03 and stroma, *P* = 0.009), VEGFA immunoscores (functionalis glands, *P* = 0.03 and stroma, *P* = 0.01) and *VEGFA* mRNA levels (*P* = 0.008) were increased in matched post-hysterectomy versus pre-hysterectomy samples. Similarly, in EC, an increase in VEGFA immunoscores (epithelial and stromal) and *VEGFA* mRNA expression was observed in the matched post-hysterectomy versus pre-hysterectomy biopsies (*P* = 0.008, *P* = 0.004 and *P* = 0.018, respectively). Full-thickness benign post-hysterectomy endometrial biopsies displayed increased *VEGFA* (*P* = 0.011) and *PR* (*P* = 0.006) mRNA expression compared with time-matched pipelle biopsies.

**LARGE SCALE DATA:**

N/A.

**LIMITATIONS, REASONS FOR CAUTION:**

This descriptive study explores the effect of pre-analytical variables on the expression of four proteins and three hypoxia-related genes in a limited number of endometrial biopsies from patients with EC and benign controls. Due to the small number, it was not possible to investigate other potential variables such as menstrual cycle phase, region-specific differences within the endometrium, grade and stage of cancer, and surgical technicalities.

**WIDER IMPLICATIONS OF THE FINDINGS:**

Careful consideration of the effects of these pre-analytical variables is essential when interpreting data relating to human endometrial biopsies. A standardized approach to endometrial tissue collection is essential to ensure accurate and clinically transferrable data.

**STUDY FUNDING/COMPETING INTEREST(S):**

The authors have no conflicts of interest to declare. The work included in this manuscript was funded by Wellbeing of Women project grants RG1073 and RG2137 (D.K.H.), Wellbeing of Women Entry-Level Scholarship ELS706 and Medical Research Council MR/V007238/1 (A.M./D.K.H.), Liverpool Women’s Hospital Cancer Charity (M.A.) and University of Liverpool (L.B., L.R. and E.N.).

WHAT DOES THIS MEAN FOR PATIENTS? Tissue biopsies from patients are vital for research, to improve our understanding of diseases and the development of treatments. Previous studies have shown that events preceding the collection of a tissue biopsy can affect the results of subsequent experiments. This is important to know, as it can affect the accuracy of the conclusions drawn from the data. This has not previously been investigated in human endometrium (the lining of the uterus).

This research was carried out on two groups of women who were having a hysterectomy for either endometrial cancer (EC) or non-cancer indications such as pelvic organ prolapse. Endometrial tissue biopsies were taken from the same patients at different time points (before or after hysterectomy) using different methods. The effect of these variables was examined by comparing the levels of a protein called hypoxia-inducible factor-1-alpha (HIF1α), which governs the tissue oxygen levels, and three proteins that are controlled by HIF1α, as well as their gene expression levels in the biopsies. The researchers found that there were distinct differences in the protein and gene expression levels, depending on the timing and type of biopsy, and the presence or absence of EC cells.

This study highlights that, as in other tissues, human endometrial biopsies are also affected by variables related to their collection, which can affect the results of downstream experiments. It is important to consider this when interpreting data from patient-derived biopsies and, where possible, to use a standardized approach to endometrial biopsy collection to improve the validity of results.

## Introduction

Human endometrial biopsies are fundamental to research in the fields of gynaecology, reproduction and cancer (Maclean *et al.*, 2020b). The endometrium is a dynamic mucosal tissue that lines the uterine cavity, consisting of lumen and gland-forming epithelium, stroma, blood vessels, lymphatics, neurones and leucocytes (Gargett *et al.*, 2016). It is organized into two functionally distinct layers, the stratum functionalis and the stratum basalis, which overlie the myometrium (Tempest *et al.*, 2018). The most distinctive feature of the human endometrium is menstruation, during which the stratum functionalis is shed and subsequently regenerated from the underlying basalis. Menstruation is a unique process, shared only by humans and upper-order primates, and is not replicated in the commonly used laboratory animal models such as rodents (Cha *et al.*, 2012). Therefore, patient-derived bio-specimens are essential in translational research (Adishesh *et al.*, 2017) to improve the current understanding of menstrual disorders, subfertility and EC. This will contribute to providing new diagnostic and prognostic indicators, and therapeutic targets (Lee *et al.*, 2015; Adishesh *et al.*, 2017; Maclean *et al.*, 2020b; Tempest *et al.*, 2020).

Data generated by patient-derived specimens have been shown to be affected by their quality, processing and storage, for a variety of human tissues (Jewell *et al.*, 2002; Espina *et al.*, 2008; Grushko *et al.*, 2022), and numerous studies have reported wide-ranging factors that influence gene and protein expression data, which are specific to bio-banking or particular to the patient (Almeida *et al.*, 2004; Ma *et al.*, 2012; Lee *et al.*, 2015; Grizzle *et al.*, 2016; Pedersen *et al.*, 2018). There is preliminary evidence that endometrial tissues obtained after hysterectomy are susceptible to changes in protein expression compared to matched pre-hysterectomy endometrial aspirates (Maiques *et al.*, 2014). However, the full effect of pre-analytical variables such as the sample collection method and processing has not yet been fully elucidated in the human endometrium. These factors need to be considered when interpreting data from patient-derived biopsies, to prevent misinterpretation of results (Adishesh and Hapangama, 2019). With the advent of molecular targeted therapies, there is an increasingly important clinical role for gene expression studies providing individualized information to guide patient therapy, giving impetus to the move towards synchronization of bio-banking standards (Sheldon *et al.*, 2011; Adishesh *et al.*, 2017; Adishesh and Hapangama, 2019).

A role for hypoxia as a normal physiological process in the cycling endometrium is supported by the presence of local tissue hypoxia at menstruation (Critchley *et al.*, 2006; Maybin *et al.*, 2011b, 2018; Cousins *et al.*, 2016). However, endometrial biopsies obtained post-hysterectomy are further subjected to ischaemia due to a compromised blood supply during the time delay between devascularization and resection of the uterus. Ischaemia has been reported to alter downstream protein and gene expression in other tissues (Atkin *et al.*, 2006; Schlomm *et al.*, 2008; Gundisch *et al.*, 2012; Grizzle *et al.*, 2016; Pedersen *et al.*, 2018). Hypoxia is also a common feature of cancers, as oxygen demand is surpassed by supply in rapidly proliferating cells (Muz *et al.*, 2015). Hypoxia-inducible factor-1-alpha (HIF1α) protein is the oxygen-responsive subunit of the heterodimeric nuclear transcription factor HIF1 that mediates the cellular response to hypoxia (Semenza, 2000). Cells constitutionally synthesize HIFα protein but, under reduced concentrations of oxygen, the HIF1α protein level increases due to its reduced degradation. HIF1 is vital for oxygen homeostasis, through increased stability (thus increased levels) of its monomers including HIF1α, which acts as a marker of cell hypoxia (Zhang and Salamonsen, 2002). It regulates the transcription of many hypoxia-related genes, including vascular endothelial growth factor A (*VEGFA*), carbonic anhydrase 9 (*CA9*) and progesterone receptor (*PR*; Henriquez *et al.*, 2017). HIF1 generates an inflammatory response and angiogenesis in response to the hypoxia through transcriptional activation of angiogenic genes, such as the downstream target and gene of interest, *VEGFA*. VEGFA is a dominant inducer of blood vessel growth, mediating angiogenesis from pre-existing vessels (Shweiki *et al.*, 1992). The enzyme CA9 is also a cellular biomarker of hypoxia, particularly in tumours, and is one of the most sensitive endogenous sensors of HIF1 activity (Kaluz *et al.*, 2009).

We conducted a study examining the differential HIF1α protein levels, and three downstream HIF1α-related genes (*VEGFA*, *CA9* and *PR*) and the levels of proteins they encode in endometrial cancer (EC) compared with benign endometrial tissue biopsies. High expression levels of PR have been suggested to be associated with favourable survival after EC (Yang *et al.*, 2014; Zhang *et al.*, 2015), hence it was selected for inclusion in this study due to its potential prognostic relevance, as well as its involvement in the regulation of HIF1α expression (Daikoku *et al.*, 2003; Song *et al.*, 2008; Habib *et al.*, 2014). The study was designed to test the hypothesis that pre-analytical variables alter downstream protein and gene expression in human endometrial biopsies.

## Materials and methods

### Ethical approval

Adult Research Ethics Committee approval for the study was obtained (19/WA/0271 and 19/SC/0449).

### Patient population

A total of 50 patients undergoing hysterectomy at a large tertiary hospital were recruited between 2017 and 2018. Each participant gave written informed consent for the use of human samples in this study. This included 22 patients undergoing hysterectomy for benign pathologies (heavy menstrual bleeding, pelvic pain, pelvic organ prolapse or simple ovarian cysts, without any known endometrial pathology). Benign samples were assigned to a menstrual cycle phase by two experienced endometrial pathologists using histological criteria as previously described and patient-reported last menstrual period date (Kamal *et al.*, 2016). A further 28 samples were obtained from patients undergoing hysterectomy as treatment for EC, none of whom had received pre-operative chemo/radiotherapy. Overall, seven patients were excluded from the study (benign group, n = 1; EC group, n = 6) due to poor-quality endometrial samples. The remaining 22 patients in the EC group included 17 cases of endometrioid adenocarcinoma (grade 1 = 8, grade 2 = 6, grade 3 = 3), three cases of carcinosarcoma, one case of clear cell carcinoma and one case of serous carcinoma.

In the benign group of 21 patients, paired endometrial biopsies (pre- and post-hysterectomy) were obtained from 17 patients, and additional myometrial biopsies (post-hysterectomy only) were obtained from four patients. From the EC group, paired endometrial biopsies were obtained from all 22 patients. See Table I and Supplementary Fig. S1 for patient demographics and cohorts.

**Table I:** Demographic data of participants.

Group	Type	Number	Age (years)	BMI (kg/m²)	Smoker (%)	Parity
**Benign**	Menstrual	1	47	35	100	3
	Proliferative	10	40 (24–47)	28 (20–37)	20	2 (0–5)
	Secretory	6	41 (34–46)	28 (21–32)	40	2 (2–5)
	Myometrium	4	43 (34–57)	24 (20–28)	0	1 (0–3)
	**All benign**	**21**	**41 (24–57)**	**26.9 (20–35)**	**23**	**2 (0–5)**
**EC**	EAC	17	68 (58–85)	35 (23–47)	28.6	2 (0–3)
	Other^*^	5	76 (72–83)	30 (19–51)	40	2 (0–3)
	**All EC**	**22**	**73 (58–85)**	**34 (19–51)**	**31.6**	**2 (0–3)**

EAC, endometrioid adenocarcinoma; EC, endometrial cancer. Data expressed as median (range).

* Other = carcinosarcoma (n = 3), serous carcinoma (n = 1), clear cell carcinoma (n = 1).

### Samples

Pre-hysterectomy samples were taken using a pipelle endometrial sampler after induction of anaesthesia in all patients, except those having a myometrial biopsy only. Post-hysterectomy samples were collected from the surgically removed uterus immediately after its removal from the patient in the operating theatre, using two methods: a pipelle sampler in both groups and a further full-thickness (FT) in only post-hysterectomy biopsies from the benign group. The FT biopsy was obtained by removing a wedge-shaped section of endometrium from the lumen to the myometrium, including functional and basal layers. FT biopsies were not obtained from the EC group to prevent disruption of the specimen prior to pathological assessment. Immediately following removal, samples were placed in neutral buffered formalin (NBF) or RNALater. RNALater was aspirated prior to storage at −80°C until RNA extraction. Samples in NBF were stored at 4°C for 24 h, and subsequently processed and impregnated in paraffin wax for long-term storage at room temperature.

Following International Federation of Gynaecology and Obstetrics guidance (Zaino *et al.*, 1995), gynaecological pathologists allocated histological descriptors for EC type and grade. Benign endometrial samples were assigned phases according to histological features and last menstrual date as described by Noyes *et al.* (1975). Haematoxylin and eosin (H&E) staining of formalin-fixed paraffin-embedded (FFPE) tissue sections was performed to assess the quality of biopsies, prior to performing experiments. Seven patients were excluded from the original cohort of 50 patients due to the following reasons: sample lacking any endometrial compartments and containing only blood/mucous or EC samples lacking cancerous epithelial cells. The remaining 43 patients were included in the study, and the groups and biopsy types can be seen in Supplementary Fig. S1.

### Quantitative real-time PCR

RNA was extracted, quantified and reverse transcribed as previously described using TRIzol^®^ Plus RNA Purification System (Life Technologies, Paisley, UK) and iScript cDNA synthesis kit (Bio-Rad, Hemel-Hempstead, UK) after DNase treatment (Promega, Southampton, UK) following the manufacturers protocol (Mathew *et al.*, 2016). cDNA (1 μg per sample) was amplified in triplicate using iTaq universal SYBR Green Supermix and CFX Connect Real-Time System (Bio-Rad, Hertfordshire, UK). Primers and reaction conditions are listed in Supplementary Table SI. Melt curves were obtained for all genes from 65°C to 95°C in 0.5°C increments for 5 seconds. Relative mRNA transcript expression for *CA9*, *VEGFA* and *PR* was calculated using the ΔΔCT method, relative to the reference genes, beta actin (ACTB) and peptidylprolyl Isomerase A (PPIA) and normalized to Ishikawa cell line expression using Bio-Rad CFX Manager (Bio-Rad, Hertfordshire, UK).

### Immunohistochemistry

FFPE tissue sections (3 µm) underwent heat-induced antigen retrieval in citrate buffer at pH 6 as previously described (Kamal *et al.*, 2016) and were immunostained with anti-human HIF1α, CA9, VEGFA and PR antibodies. Antibody details are given in Supplementary Table SII.

### Analysis of immunohistochemistry staining

Immunostaining for HIF1α, CA9 and VEGFA was assessed semi-quantitatively using a modified quickscore, by two independent observers, which encompasses both staining intensity and abundance, as previously described (Valentijn *et al.*, 2013). PR immunostaining was assessed in a similar method, using the Liverpool Endometrial Steroid Receptor Quickscore method (LESQS; Kamal *et al.*, 2016). Discrepancies between the two observers were resolved by re-evaluating the samples and agreeing on a final score. Epithelial and stromal staining, as well as nuclear and cytoplasmic staining of HIF1α, were scored separately in the basalis and functionalis of the endometrial samples, taking into account the entire sample. Poor-quality samples were excluded and in the case of samples lacking certain regions of the endometrium, only the present regions were scored.

### Statistical analyses

All statistical analyses were performed using Graphpad Prism v 5.0, employing non-parametric tests (Kruskal–Wallis and/or Mann–Whitney *U*-test or Wilcoxon signed rank test). Descriptive values are presented as median and range unless otherwise stated. Results were considered statistically significant when *P* < 0.05.

## Results

### Demographic data

The patient demographics are detailed in Table 1 and the sample group is shown in Supplementary Fig. S1. Patients with EC were significantly older than the benign group (*P* < 0.0001) and had significantly increased BMI (*P* = 0.02). There were no significant differences in smoking status or parity between the EC and benign groups. Samples from the benign group included premenopausal endometrium at different stages of the menstrual cycle, with one in the menstrual phase, 10 in the proliferative phase and 6 in the secretory phase, while in 4 cases, only myometrium was obtained. The EC group participants were all postmenopausal.

### EC is associated with increased HIF1α and CA9 immunostaining scores and increased *CA9 and VEGFA* mRNA levels

**Figure 1.**
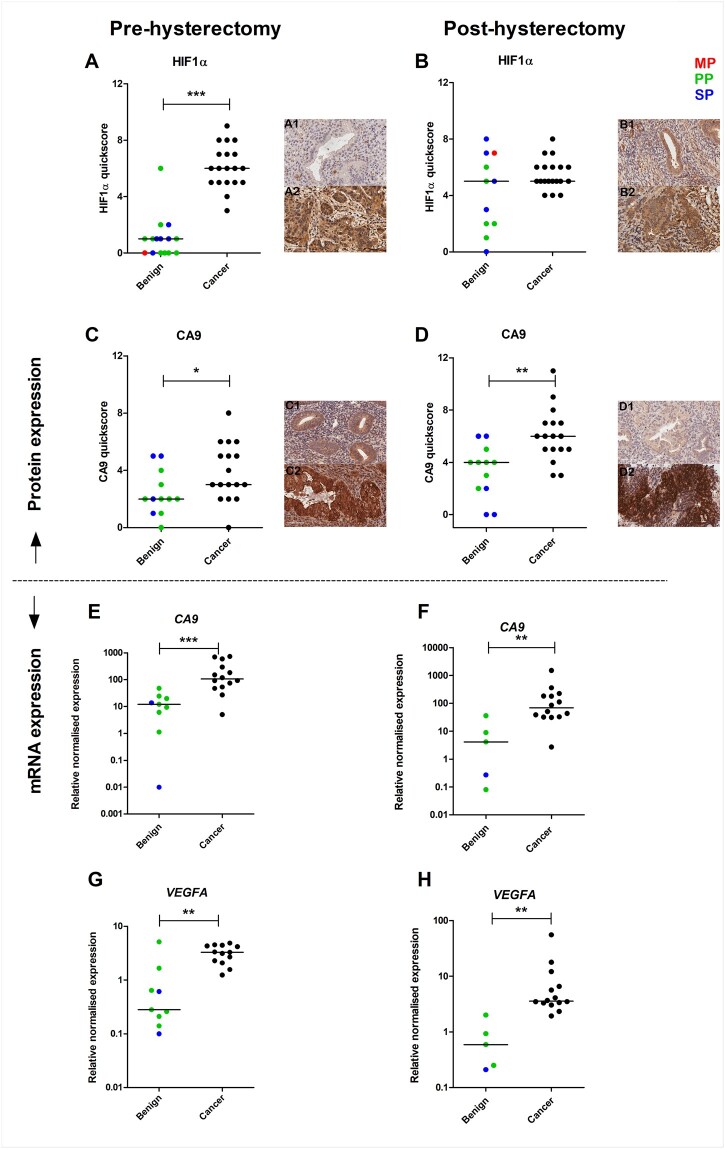
**HIF1α and VEGFA immunoscores and *VEGFA* and *CA9* mRNA expression in pre- and post-hysterectomy endometrial biopsies from endometrial cancer (EC) and benign endometrium, and representative photomicrographs illustrating the distribution of immunoreactivity for HIF1α and CA9.** Photomicrographs are high power (×40). Immunoreactivity is shown by brown positive nuclear staining. Scale bars = 60 µm in all panels. Immunoscores are modified quickscore as previously described (Valentijn *et al.*, 2013). Menstrual cycle phase of samples in the benign group is indicated by colour: red = menstrual phase (MP), green = proliferative phase (PP), blue = secretory phase (SP). (**A**) HIF1α immunoscores in pre-hysterectomy biopsies from EC compared to benign endometrium functionalis glands (FG). (**B**) HIF1α immunoscores in post-hysterectomy biopsies from EC compared to benign endometrium FG. (**C**) CA9 immunoscores in pre-hysterectomy biopsies from EC compared to benign endometrium FG. (**D**) CA9 immunoscores in post-hysterectomy biopsies from EC compared to benign endometrium FG. (**E**) *CA9* in pre-hysterectomy pipelle biopsies from EC compared to benign endometrium samples. (**F**) *CA9* in post-hysterectomy pipelle biopsies from EC compared to benign endometrium. (**G**) *VEGFA* in pre-hysterectomy pipelle biopsies from EC compared to benign endometrium. (**H**) *VEGFA* in post-hysterectomy pipelle biopsies from EC compared to benign endometrium. HIF1α, hypoxia-inducible factor-1-alpha; VEGFA, vascular endothelial growth factor A; CA9, carbonic anhydrase 9.

HIF1α immunoexpression was significantly increased in pre-hysterectomy pipelle samples from patients with EC compared with samples of benign endometrial functionalis glands (6 (6–9) n = 19 vs 1 (0–6) n = 15, *P* < 0001, respectively; Fig. 1A). No significant difference in HIF1α immunostaining was observed in post-hysterectomy pipelle biopsies between EC and benign endometrial functionalis glands (5 (4–8) n = 19 vs 5 (0–8) n = 11; Fig. 1B).

Similarly, CA9 immunoexpression was significantly increased in pre-hysterectomy pipelle samples from EC patients compared with benign endometrial functionalis glands (3 (0–8) n = 17 vs 2 (0–5) n = 12, *P* = 0.03, respectively; Fig. 1C). There was also a significant increase in CA9 immunoscores in post-hysterectomy pipelle biopsies in EC compared to benign functionalis glands (6 (3–11) n = 17 vs 4 (0–6) n = 12, *P* = 0.003, respectively; Fig. 1D). *CA9* mRNA expression was significantly increased in pre-hysterectomy pipelle samples from patients with EC compared with benign endometrial samples (106 (5.02–735.5) n = 14 vs 12.12 (0.01–47.76) n = 9, *P*=<0.001; Fig. 1E), and was also significantly increased in post-hysterectomy pipelle samples from patients with EC compared to benign endometrial samples (68.44 (2.71–1514) n = 14 vs 4.13 (0.08–36.3) n = 5, *P* = 0.008; Fig. 1F).

*VEGFA* mRNA expression was significantly increased in pre-hysterectomy pipelle samples from patients with EC compared with benign endometrial samples (3.28 (1.24–4.87) n = 13 vs 0.28 (0.1–5.12) n = 9, *P* = 0.004; Fig. 1G), and was also significantly increased in post-hysterectomy pipelle samples from patients with EC compared with benign endometrial samples (3.57 (1.93–55.41) n = 14 vs 0.59 (0.21–2.01) n = 5, *P* = 0.002; Fig. 1H). However, no significant difference in VEGFA immunoexpression was observed between EC and benign functionalis glands in pre-hysterectomy (4 (1–7) n = 17 vs 3 (0–8) n = 12, *P* = 0.34, respectively) or post-hysterectomy pipelle biopsies (6 (1–11) n = 17 vs 7 (4–10) n = 12, *P* = 0.38, respectively; data not shown).

No significant difference in *PR* mRNA expression was observed in pipelle samples from EC compared to benign endometrium obtained pre-hysterectomy (3.53 (0.13–24.52) n = 14 vs 4.8 (0.62–43.4) n = 9, respectively) or post-hysterectomy (7.56 ± 9.44 n = 14 vs 4.04 ± 2.89 n = 5, respectively; data not shown).

### *PR* mRNA expression is significantly reduced in high-grade EC samples regardless of timing of biopsy

**Figure 2.**
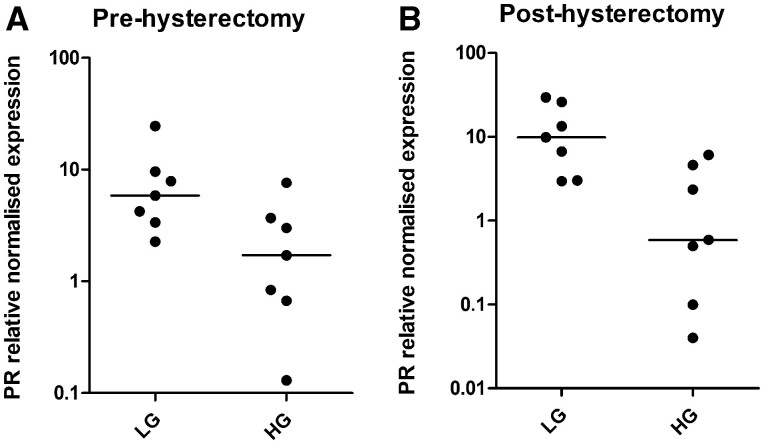
***PR* mRNA expression in low-grade (LG) and high-grade (HG) endometrial cancer (EC).** (**A**) Pre-hysterectomy pipelle biopsy in LG versus HG EC. (**B**) Post-hysterectomy pipelle biopsy in LG versus HG EC.

In EC, *PR* mRNA expression was significantly reduced in high-grade compared to low-grade EC samples, independent of the timing of when the biopsy was obtained (pre-hysterectomy: 1.71 (0.13–6.7) n = 7 vs 5.87 (2.27–24.52) n = 7, *P* = 0.026, and post-hysterectomy: 0.59 (0.04–6.08) n = 7 vs 9.88 (2.96–29.69) n = 7, *P* = 0.038; Fig. 2). No significant differences in PR immunoscores were observed between high-grade and low-grade EC in either pre- or post-hysterectomy biopsies in cancerous epithelia or stroma (Supplementary Fig. S2).

### The timing of endometrial sampling affects HIF1α protein and HIF1α-related gene and protein expression at a cellular level

The effect of time of sampling on HIF1α protein levels and gene (mRNA) expression levels of three HIF1α-related genes, *CA9*, *VEGFA* and *PR* was investigated in 39 paired (collected from the same patient) pre- and post-hysterectomy endometrial biopsies from EC (n = 22) and benign endometrial samples (n = 17).

The median time from induction of anaesthesia to pre-hysterectomy pipelle biopsy was 25 min (range: 8–98 min), and the median time between pre- and post-hysterectomy biopsies was 190 min (range: 57–270 min).

**Figure 3.**
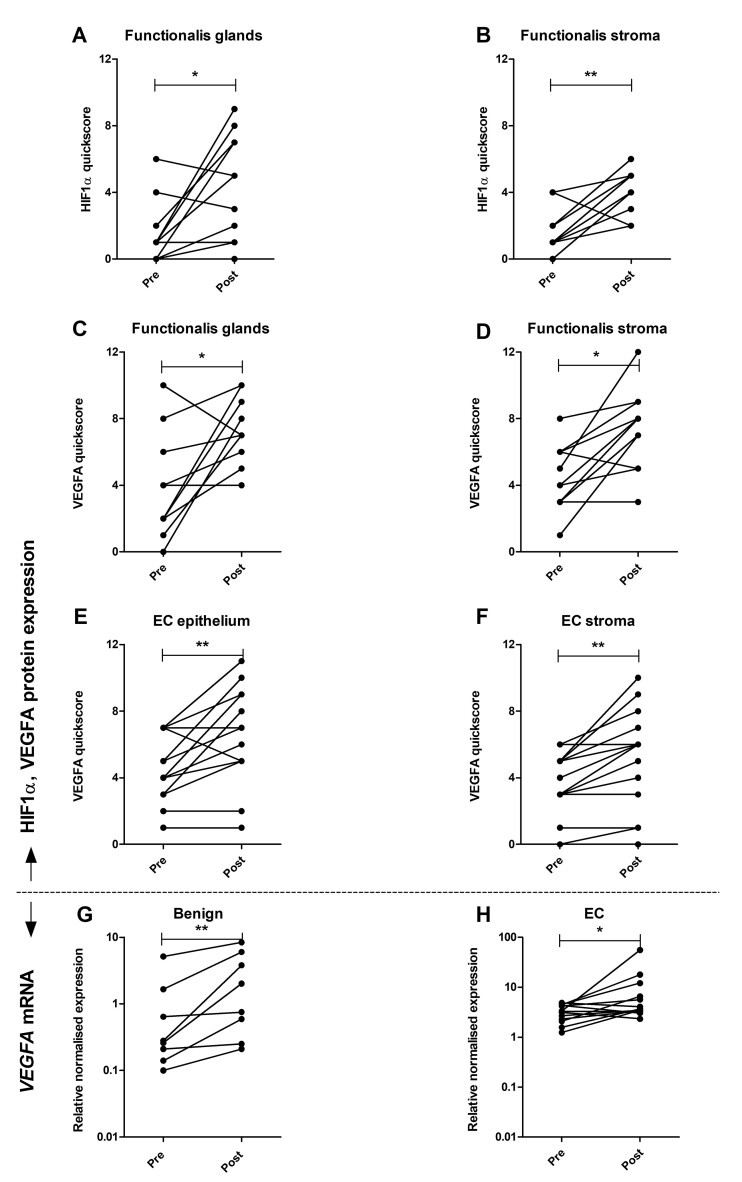
**HIF1α and VEGFA immunoscores and *VEGFA* mRNA expression in paired pre-hysterectomy (pipelle) and post-hysterectomy (pipelle and full thickness (FT)) endometrial biopsies.** Immunoscores are modified quickscore as previously described (Valentijn *et al.*, 2013). (**A**) HIF1α immunoscores in benign endometrium functionalis glands (FG) in pre- versus post-hysterectomy biopsies. (**B**) HIF1α immunoscores in benign endometrium functionalis stroma (FS) pre- versus post-hysterectomy. (**C**) VEGFA immunoscores in benign endometrium FG in pre- versus post-hysterectomy biopsies. (**D**) VEGFA immunoscores in benign endometrium FS pre- versus post-hysterectomy. (**E**) VEGFA immunoscores in EC epithelium pre- versus post-hysterectomy. (**F**) VEGFA immunoscores in EC stroma pre- versus post-hysterectomy. (**G**) *VEGFA* in benign endometrium pre- versus post-hysterectomy. (**H**) *VEGFA* in EC pre- versus post-hysterectomy biopsies. HIF1α, hypoxia-inducible factor-1-alpha; VEGFA, vascular endothelial growth factor A.

A significant increase in HIF1α immunoscores was observed in benign endometrium in post-hysterectomy compared to pre-hysterectomy samples in the functionalis glands (5 (1.25–9) n = 12 vs 1 (0–6) n = 13, *P* = 0.03, respectively; Fig. 3A), and in functionalis stroma (5 (2–6) n = 13 vs 1 (0–4) n = 13, *P* = 0.009, respectively; Fig. 3B). There was no statistically significant difference in HIF1α immunostaining between pre- and post-hysterectomy EC biopsies (Supplementary Fig. S3).

The same was observed for VEGFA protein expression, with increased immunoscores in post-hysterectomy compared to pre-hysterectomy samples in the functionalis glands (7 (4–10) n = 12 vs 3 (0–10) n = 12, *P* = 0.03, respectively; Fig. 3C), and functionalis stroma (8 (3–12) n = 12 vs 4 (1–8) n = 12, *P* = 0.01, respectively; Fig. 3D). In EC, VEGFA immunoscores also increased in the post-hysterectomy compared to pre-hysterectomy samples in both cancerous epithelial cells (6 (1–11) n = 17 vs 4 (1–7) n = 17, *P* = 0.008, respectively; Fig. 3E), and cancerous stromal compartments (6 (0–10) n = 17 vs 3.5 (0–6) n = 17, *P* = 0.004, respectively; Fig. 3F).

*VEGFA* mRNA expression was significantly increased in benign endometrial samples obtained post-hysterectomy compared to pre-hysterectomy samples (0.93 (0.21–8.38) n = 9 vs 0.28 (0.1–5.12) n = 9, *P* = 0.008, respectively; Fig. 3G). Also in EC samples, post-hysterectomy samples displayed significantly increased *VEGFA* mRNA compared to pre-hysterectomy samples (3.65 (2.33–55.41) n = 13 vs 3.28 (1.24–4.87) n = 13, *P* = 0.018, respectively; Fig. 3H).

EC samples also displayed an increase in CA9 immunoscores within the cancerous epithelial cells in post-hysterectomy compared to pre-hysterectomy samples (6 (3–11) n = 17 vs 3 (0–8) n = 17, *P* = 0.002, respectively; Supplementary Fig. S4). No other significant differences were seen in immunoscores or mRNA expression levels for *CA9* or *PR* between pre- and post-hysterectomy samples in either group (Supplementary Fig. S5).

### The sampling method employed affects downstream analysis of some endometrial markers

In benign endometrium, post-hysterectomy samples (n = 7) were obtained using two tissue harvesting methods, a pipelle sampler and a standard FT endometrial wedge biopsy, and the effect of the sampling method on protein and gene expression was assessed.

Immunoexpression of HIF1α, CA9 and VEGFA were directly compared between specific regions (functionalis and basalis) and cell types (epithelial and stromal), and no significant difference was observed between the same endometrial sub-regions in post-hysterectomy pipelle samples compared with the post-hysterectomy FT samples (Supplementary Tables SIII, SIV and SV).

**Figure 4.**
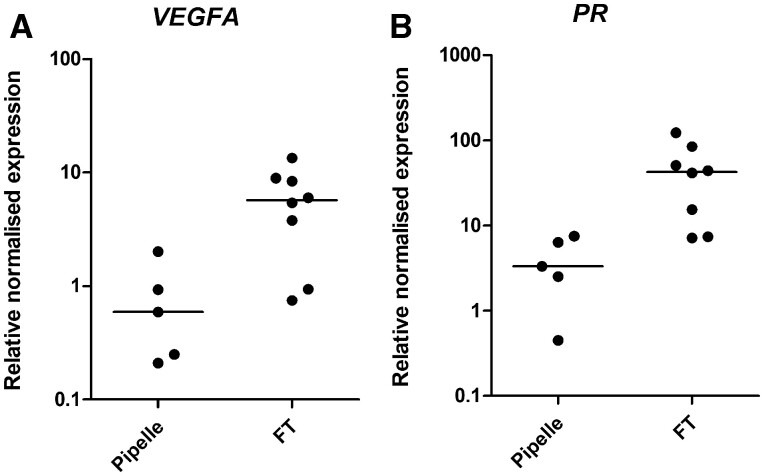
***VEGFA* and *PR* mRNA expression in benign endometrium post-hysterectomy samples.** (**A**) *VEGFA* pipelle versus full-thickness samples. (**B**) *PR* pipelle versus full-thickness samples. VEGFA, vascular endothelial growth factor A; PR, progesterone receptor.

**Figure 5.**
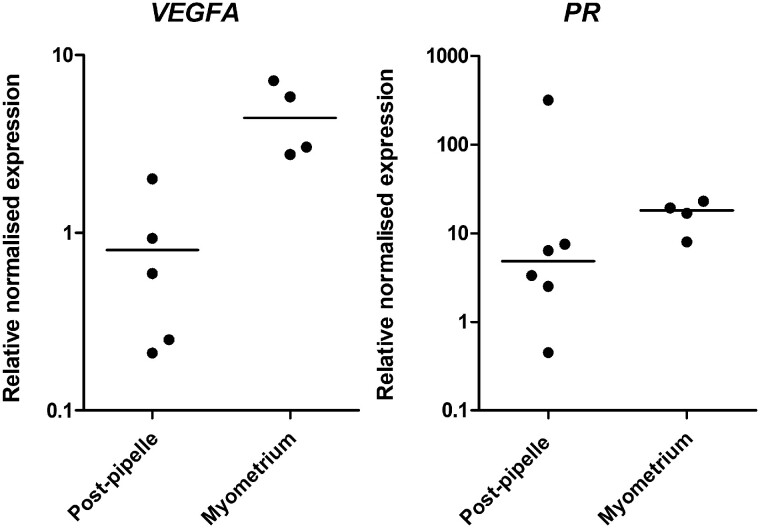
***VEGFA* and *PR* mRNA expression in benign endometrial pipelle biopsy obtained post-hysterectomy compared to post-hysterectomy myometrial biopsy.** (**A**) *VEGFA*. (**B**) *PR*. VEGFA, vascular endothelial growth factor A; PR, progesterone receptor.

Significant increases in *VEGFA* and *PR* mRNA expression were observed in the post-hysterectomy FT samples compared to time-matched pipelle biopsies in benign endometrium (5.7 (0.75–13.4) n = 8 vs 0.59 (0.21–2.01) n = 5, *P* = 0.011, and 42.85 (7.19–122.7) n = 8, vs 3.34 (0.45–7.54) n = 5, *P* = 0.006, respectively; Fig. 4). FT endometrial samples often also contain the underlying myometrium. Therefore, to investigate if this observed increase in was due to myometrial contamination, we compared *VEGFA* and *PR* mRNA expression between time-matched post-hysterectomy myometrial samples and endometrial pipelle samples (which do not contain any myometrium). Significantly higher *VEGFA* mRNA levels were seen in myometrial biopsies compared to pipelle biopsies (4.43 (2.76–7.15) n = 4, vs 0.59 (0.21–2.01) n = 5, *P* = 0.0159; Fig. 5). A non-significantly higher *PR* mRNA level was observed in post-hysterectomy myometrial biopsies compared to pipelle biopsies (18.11 (7.99–23.01) n = 4 vs 4.8 (0.45–319.6) n = 6, *P* = 0.114; Fig. 5).

## Discussion

This study examines the effect of pre- or post-hysterectomy timing and sampling method of endometrial biopsies on the protein and mRNA expression levels of markers of hypoxia in paired endometrial biopsies. The quality of endometrial biopsy on generated data was assessed by direct visual assessment of the H&E stained tissue to determine the inclusion of different cell types and all endometrial anatomical regions in the biopsy.

The observed increase in markers of hypoxia in time-matched biopsies from patients with EC compared to benign endometrium highlights that potential studies comparing endometrial biopsies from these two groups must consider the effect of disease status and ischaemia on expression of downstream markers of interest. In benign endometrium, the increase in HIF1α protein and VEGFA protein and gene expression in post-hysterectomy samples compared to pre-hysterectomy samples indicates exposure of tissues to warm ischaemia intra-operatively, which was particularly evident in the functionalis glands and stroma. Surprisingly, CA9 protein and gene expression were not significantly different in post-hysterectomy benign endometrial samples, which may be due to the duration of time that the tissues were exposed to warm ischaemia. In a similar study in colorectal tissues, Atkin *et al.* (2006) reported an increase in *CA9* expression after at least 4 h of devascularization, and the average time between clamping uterine vessels and resection of the uterus in a hysterectomy is typically less than this (Cassling *et al.*, 2019), with a median time of 190 min in this study. Controlling warm ischaemia time for the purpose of preserving surgical specimen quality is not feasible, as there are many intra-operative variables which affect the duration of the operation such as extent of underlying disease, previous surgery, surgical technique and other surgical complexities. Therefore, these effects should be taken into account when interpreting results of protein and gene expression analyses in a post-hysterectomy biopsy, and thus a standardized approach to endometrial tissue collection is essential to ensure robust interpretation of generated data.

In benign endometrium, post-hysterectomy samples are typically collected as either pipelle or FT biopsies. A recent review of human endometrial biopsies highlighted that an FT sample contains functionalis and basalis of the endometrium and underlying myometrium, whereas the pipelle sampler will mostly contain functionalis only, depending on the technique and phase of menstrual cycle (Maclean *et al.*, 2020b). Interestingly, the significant increase in *VEGFA* mRNA expression in post-hysterectomy compared to pre-hysterectomy biopsies in benign endometrium was lost when only pipelle samples were considered. Furthermore, HIF1α, CA9 and VEGFA protein levels were not significantly different when the same endometrial regions (functional glands and stroma) were compared in time-matched post-hysterectomy pipelle compared to FT biopsies. Therefore, we postulate that the observed differential expression of *VEGFA* in post-hysterectomy benign endometrium is due to the presence of cells from different endometrial regions (functionalis and basalis) in FT samples. Furthermore, samples containing only myometrium demonstrated significantly high *VEGFA* mRNA expression levels when compared with time-matched pipelle biopsies, suggesting that the observed increase in *VEGFA* in post-hysterectomy samples is at least partly due to the myometrial contribution. The healthy myometrium is well vascularized and therefore highly sensitive to hypoxia, explaining the possible high expression of *VEGFA* during warm ischaemia (Harrison-Woolrych *et al.*, 1995). This should be considered when analysing FT endometrial biopsies as a whole, in gene expression and proteomic experiments. Methods allowing for region separation within tissues, such as immunostaining methods, laser capture microdissection or spatial transcriptomic analysis, will overcome this impediment.

A good-quality pipelle endometrial biopsy contains the luminal epithelium, functional glands and functional stroma in ample quantities, and in addition to those, a good-quality FT endometrial biopsy includes the basal glands and basal stroma. Endometrial biopsies may lack one or more of these cellular compartments, which affects mRNA expression and downstream analysis (Evans *et al.*, 2014; Maclean *et al.*, 2020b). This variable needs to be considered particularly when endometrial biopsies are taken as a whole.

In EC samples, an increase in CA9 protein and VEGFA protein and mRNA expression was observed in post-hysterectomy compared to pre-hysterectomy pipelle biopsies, with no change in *CA9* mRNA expression, or HIF1α protein expression. Several studies have previously suggested warm ischaemia causes significant changes in gene expression in various cancer tissues (Huang *et al.*, 2001; Schlomm *et al.*, 2008; Liu *et al.*, 2013), although this was not reflected in our results. This could be due to the small sample size in this study; however, despite the aberrant vascularization through angiogenesis, tumours are relatively hypoxic when they outgrow their oxygen supply (Muz *et al.*, 2015; Petrova *et al.*, 2018). Thus, no further significant influence on the existing hypoxia markers may be seen with post-hysterectomy sampling.

Overall, loss of *PR* in high-grade EC samples was independent of warm ischaemia, which could be inferred as validation of current bio-banking practices. The same was not observed for PR protein levels, with no significant differences between high- and low-grade EC either in pre- or post-hysterectomy samples. However, the sample size of high-grade EC was notably smaller in the immunohistochemistry experiments.

This study investigates the effect of warm ischaemia, by exploring the effect of the length of intra-operative time an endometrial tissue sample is exposed to ischaemia, in the context of four and three hypoxia-related proteins and genes, respectively. A preferred approach recommended for further research in this area would be to measure the expression of a larger number of genes. With a limited number of cases in this study, we were also unable to draw comparisons and analyse alternative putative pre-analytical variables, particularly including grade and stage of cancer, menstrual cycle phase (Maybin *et al.*, 2018), patient co-morbidities and mode of surgery. There are region-specific differences in cellular phenotype of the endometrium throughout the menstrual cycle (Valentijn *et al.*, 2013; Hapangama *et al.*, 2015; Maclean *et al.*, 2020a), and the subsequent impact on research derived from endometrial biopsies obtained during different phases has already been established (Maclean *et al.*, 2020b). There are hypoxia-related physiological changes that occur in the cycling endometrium, which undoubtedly regulate downstream gene and protein expression depending on menstrual cycle phase (Maybin *et al.*, 2011a,b, 2018), To account for this, matched endometrial biopsies were used to examine the variation in hypoxia-related markers at different time points, using different sampling methods. However, for the comparison between benign and EC samples, unpaired benign endometrial biopsies at different menstrual cycle phases were compared to mostly postmenopausal EC samples. Menstrual cycle phase is expected to affect the expression of hypoxia-related markers in the benign group; however, due to small sample sizes, we did not observe any significant differences in our cohort. Future studies in this area would benefit from larger sample sizes to enable examination of temporal differences related to warm hypoxia across the menstrual cycle. An increase in hypoxia-related proteins and genes was seen in the EC samples, compared to the benign samples, despite the differences in menopausal status between groups. In other studies, benign postmenopausal endometrium has been shown to exhibit low levels of markers of hypoxia, HIF1α and CA9 (Horrée *et al.*, 2007), which suggests that the results indicating increased hypoxia in EC samples in this study are due to a true physiological effect of disease status on the endometrium.

It is evident that pre-analytical variables such as sampling method, pre/post-hysterectomy timing of sampling and proportion of endometrial regions within the sample obtained can affect downstream analysis in the human endometrium. Careful consideration of pre-analytic variables is thus essential to produce accurate and clinically transferrable data derived from endometrial biopsies.

## Supplementary data

Supplementary data are available at *Human Reproduction Open* online.

## Data availability

The data underlying this article will be shared on reasonable request to the corresponding author.

## Supplementary Material

hoac026_Supplementary_Data
