# Supplementary material for: The effect of pre-analytical variables on downstream application and data analysis of human endometrial biopsies
Source: Hum Reprod Open. 2022 Jun 13;2022(3):hoac026. doi: 10.1093/hropen/hoac026 (PMC9240853; doi:10.1093/hropen/hoac026)
Supplement: hoac026_Supplementary_Data [file hoac026_supplementary_data.docx]

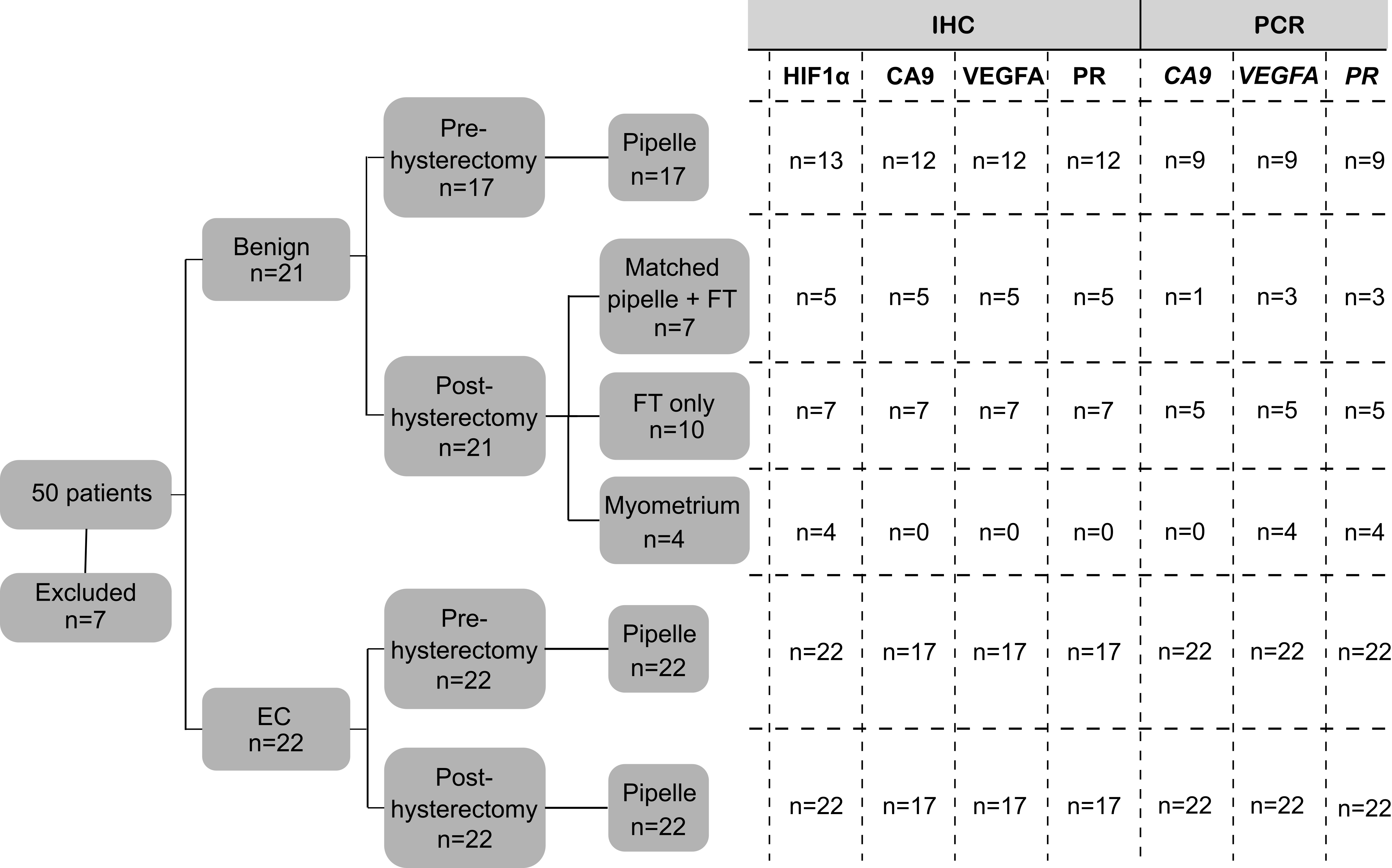


**Supplementary Figure S1.** Schematic representation of patient samples obtained and subsequent numbers in each experimental group. Benign, benign endometrium; EC, endometrial cancer; FT, full-thickness endometrial biopsy; IHC, immunohistochemistry; PCR, quantitative real-time polymerase chain reaction.


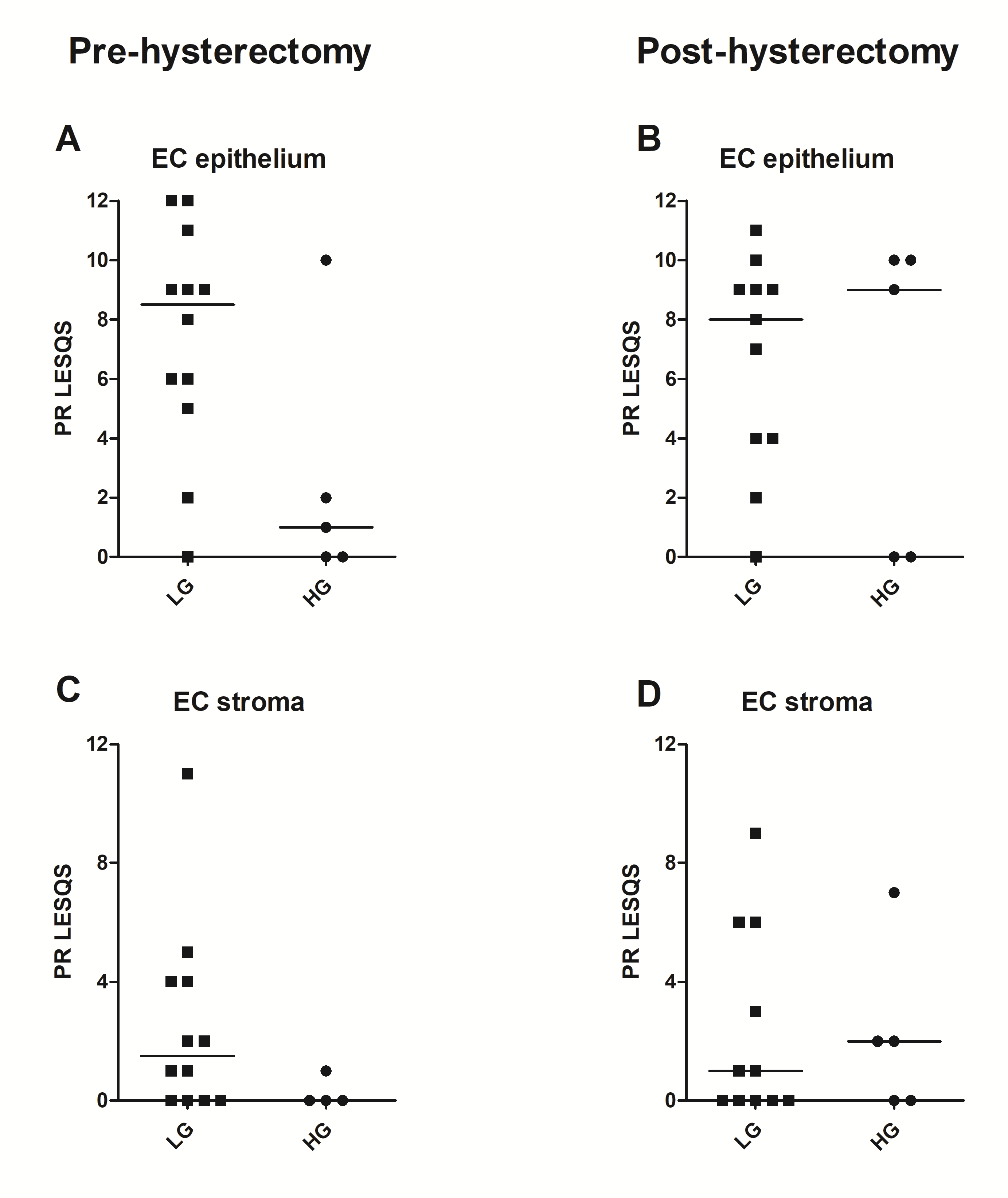


**Supplementary Figure S2**. PR immunoscores in low-grade (LG) and high-grade (HG) endometrial cancer (EC). **(A)** Pre-hysterectomy pipelle biopsy in LG vs HG EC epithelium (8.5 (0-12) n=12, vs 1 (0-10) n=5, p=0.723, respectively) **(B)** Post-hysterectomy pipelle biopsy in LG vs HG EC epithelium (8 (0-11) n=11, vs 9 (0-10) n=5, p=0.954, respectively). **(C)** Pre-hysterectomy pipelle biopsy in LG vs HG EC stroma (1.5 (0-11) n=12 vs 0 (0-1) n=4, p=0.112, respectively)**. (D)** Post-hysterectomy pipelle biopsy in LG vs HG EC stroma (1 (0-9) n=11 vs 2 (0-7) n=5, p=0.906, respectively).


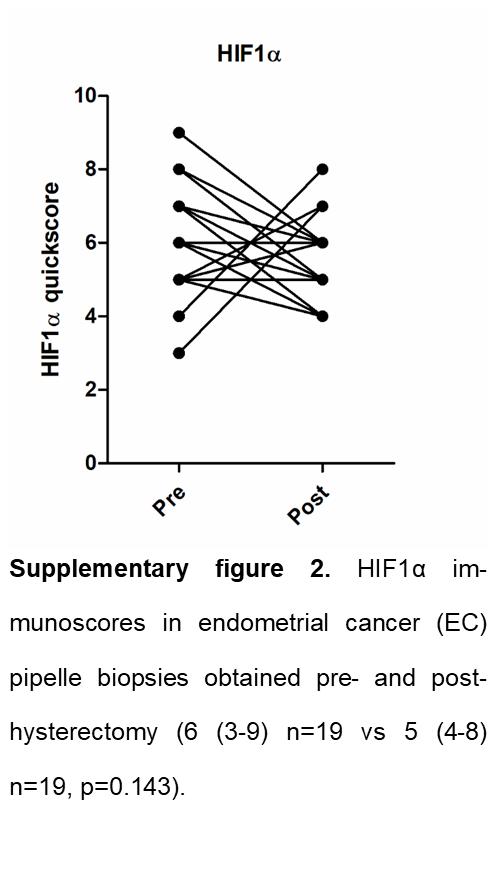


**Supplementary Figure S3**. HIF1α immunoscores in endometrial cancer (EC) pipelle biopsies obtained pre- and post- hysterectomy (6 (3-9) n=19 vs 5 (4-8) n=19, p=0.143).


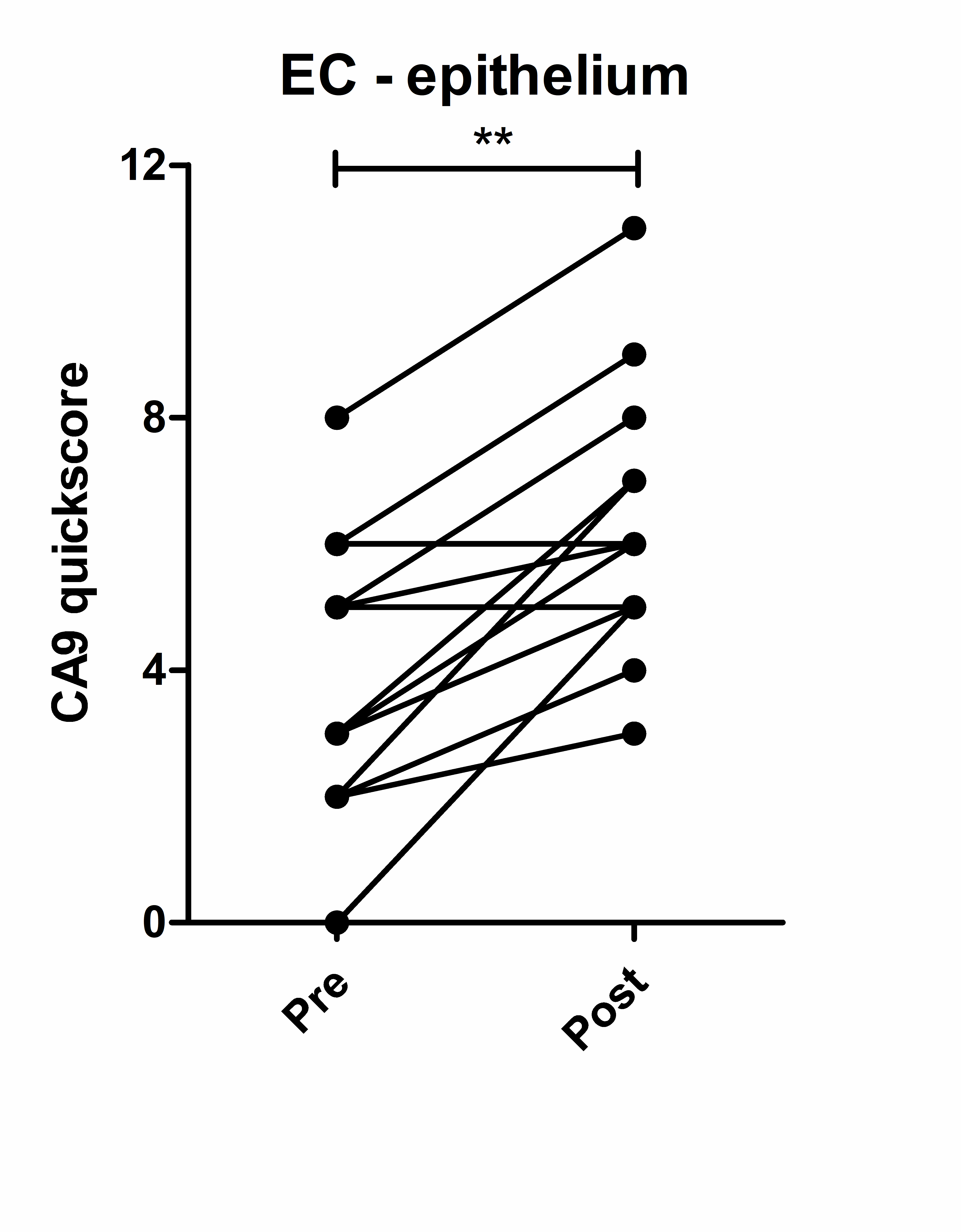


**Supplementary Figure S4**. CA9 immunoscores in endometrial cancer (EC) epithelial cells obtained pre- and post- hysterectomy (3 (0-8) n=17, vs 6 (3-11) n=17, p=0.002).

**Supplementary Figure S5**. CA9 and PR immunoscores and mRNA expression from benign endometrium and endometrial cancer (EC) samples obtained pre-hysterectomy (pipelle) and post-hysterectomy (pipelle and full-thickness). **(A)** CA9 immunoscores in functional glands (FG) of matched benign endometrial biopsies obtained pre- and post-hysterectomy (p=0.11). **(B)** CA9 immunoscores in functional stroma (FS) of matched benign endometrial biopsies obtained pre- and post-hysterectomy (p=0.4). **(C)** *CA9* mRNA expression in matched benign endometrial biopsies obtained pre- vs post-hysterectomy (p=0.91). **(D)** CA9 immunoscores in stromal compartment of matched EC samples obtained pre- vs post-hysterectomy (p=0.09). **(E)** *CA9* mRNA expression in matched EC biopsies obtained pre- vs post-hysterectomy (p=0.1). **(F)** PR immunoscores in FG of matched benign endometrial biopsies obtained pre- and post-hysterectomy (p=0.39). **(G)** PR immunoscores in FS of matched benign endometrial biopsies obtained pre- and post-hysterectomy (p=0.12). **(H)** *PR* mRNA expression in matched benign endometrial biopsies obtained pre-hysterectomy compared to post- hysterectomy (p=0.074). **(I)** PR immunoscores in cancerous epithelial compartment of matched EC samples obtained pre- vs post-hysterectomy (p=1.0). **(J)** PR immunoscores in cancerous stromal compartment of matched EC samples obtained pre- vs post-hysterectomy (p=0.5). **(K)** *PR* mRNA expression in matched EC biopsies obtained pre-hysterectomy compared to post-hysterectomy (p=0.433).


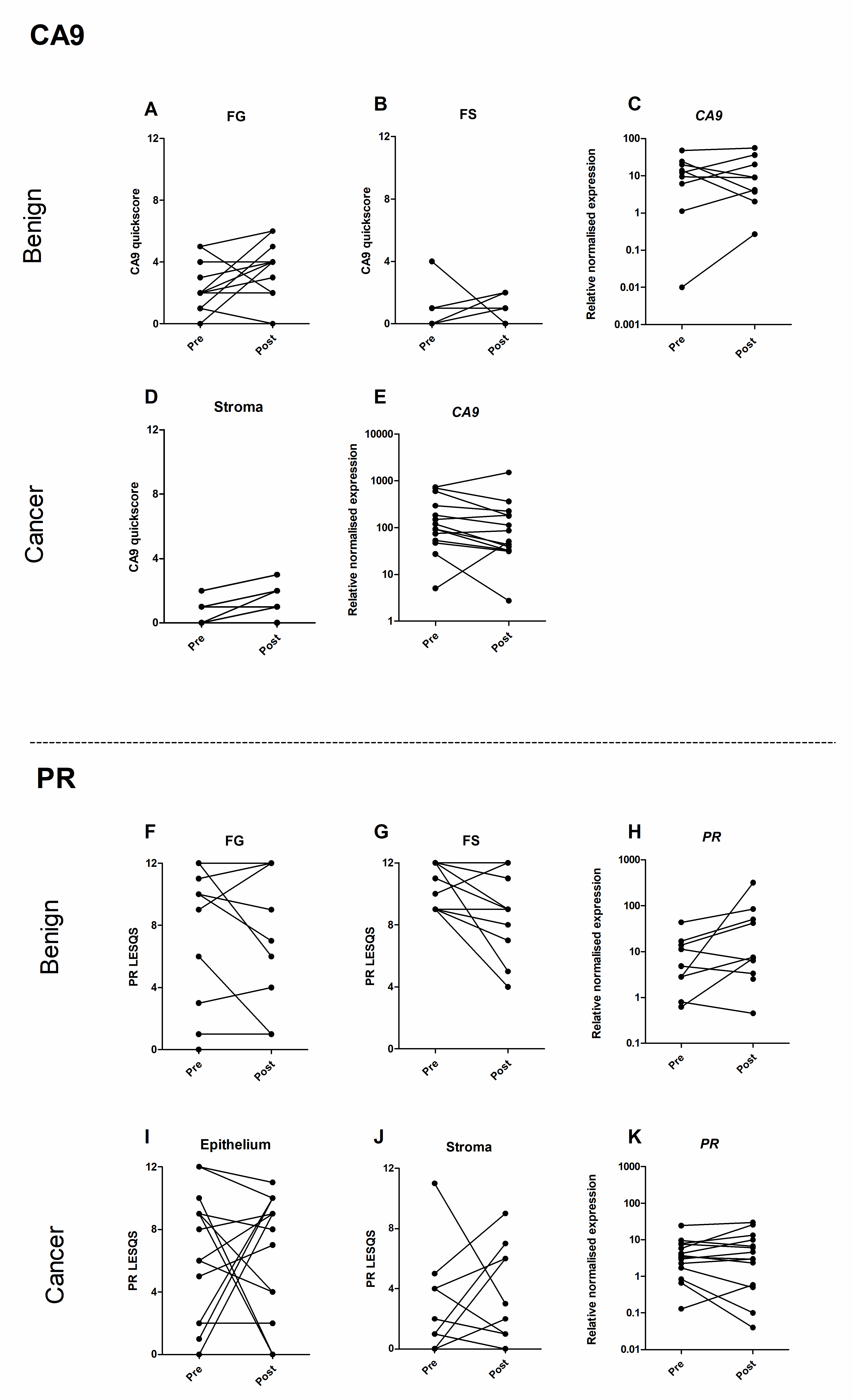


**Supplementary Table SI: Primer sequences and qPCR conditions.**

| **Gene** | **qPCR primer sequence** | **Initial denature** | **Cycle conditions*** | **Company** |
| --- | --- | --- | --- | --- |
| *CA9* | Unique Assay ID: qHsaCID0017667 RefSeq: NC_000009.11, NG_011620.1, NT_008413.18 | 95°C 2min | 95°C 5s  60°C 30s | Bio-Rad^1^ |
| *VEGFA* | Unique Assay ID: qHsaCED0043454 RefSeq: NC_000006.11, NG_008732.1, NT_007592.15 | 95°C 2min | 95°C 5s  60°C 30s | Bio-Rad^1^ |
| *PR*  (47) | F: 5’CAGTGGGCGTTCCAAATGA3’  R: 5’TGGTGGAATCAACTGTATGTCTTGA3' | 95°C 6min | 95°C 10s  60°C 20s  72°C 30s | Sigma^2^ |
| *ACTB*  (48) | F: 5’-GTACGCCCAACACAGTGCTG-3'  R: 5’GCTGGAAGGTGGACAGCGA-3' | 95°C 10min | 98°C 5s  95°C 10s  60°C 30s | Sigma^2^ |
| *PPIA*  (49) | F: 5’-AGACAAGGTCCCAAAGAC-3'  R: 5’-ACCACCCTGACACATAAA-3' | 95°C 2min | 95°C 5s  60°C 30s | Sigma^2^ |

Abbreviations: PPIA, peptidylprolyl isomerase A; ACTB, beta-actin; PR, progesterone receptor; VEGFA, vascular endothelial factor A; CA9, carbonic anhydrase 9. *40 cycles per gene.
^1^ Hertfordshire, UK. ^2^ Dorset, UK.

**Supplementary Table SII: Primary antibodies and their immunohistochemistry conditions**

| **Target** | **Host species** | **Clone** | **Supplier** | **HIAR* (min)** | **Dilution** | **Incubation conditions** | |
| --- | --- | --- | --- | --- | --- | --- | --- |
|  |  |  |  |  |  | Time (hour) | Temp (°C) |
| HIF1α | Mouse mAb | Ab16066 | Abcam^1^ | 2 | 1:400 | 20 | 4 |
| CA9 | Rabbit pAb | Ab15086 | Abcam^1^ | 2 | 1:400 | 20 | 4 |
| VEGFA | Mouse mAb | Ab1316 | Abcam^1^ | 2 | 1:800 | 20 | 4 |
| PR | Mouse mAb | PgR 636 | DAKO^2^ | 2 | 1:1000 | 0.5 | 18 |
| *Heat induced antigen retrieval by pressure cooking in citrate buffer pH 6 (Hapangama et al, 2012). ^1^ Ely, Cambridgeshire, UK ; ^2^ Cambridge, UK. | | | | | | | |

**Supplementary Table SIII:** Region specific HIF1α immunoscores in post-hysterectomy benign endometrial pipelle and full-thickness biopsies.

|  | **Sample type (n)** | **Median (range)** | **P value** |
| --- | --- | --- | --- |
| Functionalis glands | Pipelle (5) | 5 (3-9) | 0.278 |
|  | Full-thickness (11) | 5 (0-8) |  |
| Functionalis stroma | Pipelle (5) | 5 (2-6) | 0.908 |
|  | Full-thickness (11) | 5 (2-6) |  |

**Supplementary Table SIV:** Region specific VEGFA immunoscores in post-hysterectomy benign endometrial pipelle and full-thickness biopsies.

|  | **Sample type (n)** | **Median (range)** | **P value** |
| --- | --- | --- | --- |
| Functionalis glands | Pipelle (5) | 5 (5-7) | 0.172 |
|  | Full-thickness (11) | 7 (4-10) |  |
| Functionalis stroma | Pipelle (5) | 6 (5-8) | 0.25 |
|  | Full-thickness (11) | 8 (3-12) |  |

**Supplementary Table SV:** Region specific CA9 immunoscores in post-hysterectomy benign endometrial pipelle and full-thickness biopsies.

|  | **Sample type (n)** | **Median (range)** | **P value** |
| --- | --- | --- | --- |
| Functionalis glands | Pipelle (5) | 2 (2-4) | 0.713 |
|  | Full-thickness (11) | 4 (0-6) |  |
| Functionalis stroma | Pipelle (5) | 0 (0-1) | 0.346 |
|  | Full-thickness (11) | 1 (0-2) |  |
